# Supplementary material for: The Genus Miconia Ruiz & Pav. (Melastomataceae): Ethnomedicinal Uses, Pharmacology, and Phytochemistry
Source: Molecules. 2022 Jun 27;27(13):4132. doi: 10.3390/molecules27134132 (PMC9267935; doi:10.3390/molecules27134132)
Supplement: Supplementary file 1 [file molecules-27-04132-s001.zip › molecules-1765101-supplementary.pdf]

Supplementary Material

Table S1. Compounds identified in the genus *Miconia* Ruiz & Pav. and their respective biological activities.

Compounds	Structure	PubChem	Species	Plant Organ	Biological Activity	References
**Alkaloids**						
Glycine betaine	1	*CID 21872856*	*M. fallax*<br>*M. rufescens*<br>*M. stenostachya*	Aerial parts	-	[60]
Isomer of 5- carboxylyaloside	2	-	*M. latecrenata*	Leaves	Antiplasmodial	[16]
Isomer of 5- carboxystrictosidin	3	-	*M. latecrenata*	Leaves	Antiplasmodial	[16]
Isomer of cinnamoyl-4″-hydroxy-3″-methoxy-lyaloside	4	-	*M. latecrenata*	Leaves	Antiplasmodial	[16]
Isomer of lyaloside	5	*CID 11092621*	*M. latecrenata*	Leaves	Antiplasmodial	[16]
Lyalosidic acid	6	*CID 10391678*	*M. latecrenata*	Leaves	Antiplasmodial	[16]
Strictosidine	7	*CID 161336*	*M. latecrenata*	Leaves	Antiplasmodial	[16]
Strictosidinic acid	8	*CID 21586927*	*M. latecrenata*	Leaves	Antiplasmodial	[16]
Trans-4- hydroxy-methyl-*D*-proline	9	*CID 12313446*	*M. dispar*<br>*M. fallax*<br>*M. rufescens*<br>*M. stenostachya*	Aerial parts	-	[60]
**Flavonoids**						
(−)-epicatechin	10	*CID 72276*	*M. rubiginosa*	Leaves	-	[61]
5,6,7-trihydroxy- 4′-methoxyflavone	11	-	*M. ferruginata*	Leaves	Insecticide	[26]
5,7,4′-trihydroxy-6,8-dimethoxyflavone	12	-	*M. ferruginata*	Leaves	Insecticide	[26]
5-hydroxy-4′,7-dimethoxyflavone-(6→6″) -5″-hydroxy-3‴,4‴,7″-trimethoxyflavone	13	-	*M. cabucu*	Aerial parts	-	[62]
5-Hydroxy-4',7-dimethoxy-8-methylflavone	14	*CID 91213897*	*M. ferruginata*	Leaves	Insecticide	[26]
Matteucinol 7-O-β-d-apiofuranosyl-(1 → 6)-β-d-glucopyranosyl	15	*CID 42607943*	*M. prasina*	Stem	-	[63]
Apigenin-7-O-glucoside	16	*CID 44257792*	*M. alypifolia*	Leaves	Antioxidant	[49]
Astragalin	17	*CID 5282102*	*M. albicans*	Fruits	Antioxidant and anti-inflammatory	[13]
Cyanidin-3- *O*-rutinoside	18	*CID 441674*	*M. albicans*	Fruits	Antioxidant and anti-inflammatory	[13]
Demethoxymatteucinol	19	*CID 180550*	*M. prasina*	Stem	-	[63]
Farrerol	20	*CID 91144*	*M. prasina*	Stem	-	[63]
Farrerol 7-*O*-*β*-D-apiofuranosyl(1→6)-*β*-D-glucopyranoside	21	-	*M. trailii*	Aerial parts	-	[64]
Favanone glycoside 7-O-β-D-apiofuranosyl-(1 → 6)-β-D-glucopyranosyldemethoxymatteucinol (miconioside C)	22	-	*M. prasina*	Stem	-	[63]
Hyperoside	23	*CID 5281643*	*M. chamissois*	Leaves	Antimicrobial, cytotoxic and Antioxidant	[6]
Isoquercitrin	24	*CID 5280804*	*M. albicans*	Leaves	Anti-inflammatory	[32]
Isorhamnetin	25	*CID 5281654*	*M. latecrenata*	Leaves	Antibacterial	[22]
Isovitexin	26	*CID 162350*	*M. chamissois*	Leaves	Antimicrobial, cytotoxic and Antioxidant	[6]
Kaempferol 3-*O*-α-L-arabinopyranoside	27	*CID: 5481882*	*M. albicans*	Leaves	Antidiabetic	[65]

Kaempferol	28	*CID 5280863*	*M. albicans*	Fruits	Antioxidant and anti-inflammatory	[13]
			M. burchellii.	Leaves	Cytotoxic	[55]
Kaempferol-3-O-diglucoside	29	*CID 21722032*	*M. alypifolia*	Leaves	Antioxidant	[49]
Kaempferol-3-O-*D*-galactoside	30	*CID 5488283*	*M. alypifolia*	Leaves	Antioxidant	[49]
kaempferol-3-O-β -glucopyranoside	31	-	*M. burchellii.*	Leaves	Cytotoxic	[55]
Kaempferol-3-O-β-D-(6″-coumaroyl)-glucopyranoside	32	-	*M. cabucu*	Aerial parts	-	[62]
Kaempferol-3-O-β-galactopyranoside	33	-	*M. rubiginosa*	Leaves	-	[61]
			M. burchellii.		Cytotoxic	[55]
Kaempferol-O-pentoside	34	*CID 14749097*	*M. albicans*	Leaves	Anti-inflammatory	[32]
Leucoanthocyanidin	35	*CID 124037363*	*M. albicans*	Leaves	Anti-inflammatory	[12]
Matteucinol 7-O-*α*-*L*-arabinopyranosyl(1→6)-β-*D*-glucopyranoside – (Miconioside A)	36	*CID 11050315*	*M. trailii*	Aerial parts	-	[64]
Matteucinol 7-O-β-apiofuranosyl (1 → 6)-β-glucopyranoside	37	*CID 42607943*	*M. albicans* *M. chamissois* *M. trailli*	Aerial parts	-	[64,66]
Matteucinol	38	*CID 160490*	*M. trailii*	Aerial parts	-	[64]
			M. prasina	Stem		[63]
			M. chamissois	Leaves	Angiogenic	[54]
Mattucinol-7-O-[4′ ′,6′ ′-di-O-galloyl]-β-*D*-glucopyranoside	39	*CID 42607945*	*M. myriantha*	Aerial parts	-	[67]
Mattucinol-7-O-[4′ ′,6′ ′-O-(S)-hexahydroxydiphenoyl]-β-*D*-glucopyranoside	40	-	*M. myriantha*	Aerial parts	-	[67]
Mattucinol-7-O-β-*D*-glucopyranoside	41	-	*M. myriantha*	Aerial parts	-	[67]
Mearnsetin 3-*O*-*α*-L-rhamnopyranoside	42	-	*M. albicans*	Leaves	Antidiabetic	[65]
Miconioside B	43	*CID 42607900*	*M. albicans* *M. chamissois*	Leaves	-	[66]
			M. prasina	Stem		[63]
Myricetin 3-galactoside	44	*CID 5491408*	*M. albicans*	Fruits	Antioxidant and anti-inflammatory	[13]
Myricetin-3-O- *α*-L-rhamnopyranoside	45	-	*M. cabucu*	Aerial parts	-	[62]
			M. albicans	Leaves	Antidiabetic	[65]
Myricetin-O-hexoside	46	*CID 12311099*	*M. albicans*	Leaves	Anti-inflammatory	[32]
Quercetin 3-*O*-(2″-galloyl)-*α*-L-rhamnopyranoside	47	CID 44259259	*M. albicans*	Leaves	Antidiabetic	[65]
Quercetin glycocoumarate	48	-	*M. albicans*	Fruits	Antioxidant and anti-inflammatory	[13]
Quercetin glycogallate	49	-	*M. albicans*	Fruits	Antioxidant and anti-inflammatory	[13]
Quercetin	50	*CID 5280343*	*M. chamissois*	Leaves	-	[66]
			M. albicans	Leaves	Antioxidant	[51]
					Anti-inflammatory	[12]
						[32]
				Fruits	Antioxidant and anti-inflammatory	[13]
			M. latecrenata	Leaves	Antibacterial Antimutagênica	[16]
					Antiplasmodial	[16]
Quercetin-3-O- *α* -L-rhamnopyranoside	51	-	*M. cabucu*	Aerial parts	-	[62]

Quercetin-3-O- β-D-glucopyranoside	52	-	*M. cabucu*	Aerial parts	-	[62]
Quercetin-3-O-arabinoside	53	*CID 12309865*	*M. albicans*	Fruits	Antioxidant and anti-inflammatory	[13]
Quercetin-3-O-galactoside	54	-	*M. alypifolia*	Leaves	Antioxidant	[49]
			M. albicans	Fruits	Antioxidant and anti-inflammatory	[13]
Quercetin-3-O-glucoside	55	*CID 25203368*	*M. albicans*	Leaves	Antioxidant	[51]
Quercetin-3-O-rhamnoside	56	*CID 5353915*	*M. latecrenata*	Leaves	Antibacterial	[47]
Quercetin-3-O-*α*-*D*-arabinopyranoside	57	*CID 44259270*	*M. rubiginosa*	Leaves	-	[61]
Quercetin-3-O-*α*-*L*-rhamnopyranoside	58	*CID 6325794*	*M. rubiginosa*	Leaves	-	[61]
Quercetin-3-O-*α*-rhamnopyranosil-(1→4)-O-β-galactopyranoside	59	-	*M. rubiginosa*	Leaves	-	[61]
Quercetin-3-O-β-*D*-arabinofuranoside	60	*CID 12047348*	*M. rubiginosa*	Leaves	-	[61]
Quercetin-3-O-β-galactopyranoside	61	-	*M. rubiginosa*	Leaves	-	[61]
Quercetin-7-O-glucoside	62	*CID 5381351*	*M. albicans*	Fruits	Antioxidant and anti-inflammatory	[13]
Quercetin-O-galloyl-hexoside	63	-	*M. albicans*	Leaves	Anti-inflammatory	[32]
Quercetin-O-galloyl-pentoside	64	-	*M. albicans*	Leaves	Anti-inflammatory	[32]
Quercetin-O-pentoside	65	-	*M. albicans*	Leaves	Anti-inflammatory	[32]
Quercitrin	66	*CID 5280459*	*M. latecrenata*	Leaves	Antiplasmodial	[16]
			M. albicans	Fruits	Antioxidant and anti-inflammatory	[13]
Rutin	67	*CID 5280805*	*M. chamissois*	Leaves	-	[66]
					Antioxidant	[51]
					anti-inflammatory	[12]
			M. albicans			[32]
				Fruits	Antioxidant and anti-inflammatory	[13]
			M. chamissois	Leaves	Antimicrobial, cytotoxic and Antioxidant	[6]
Vitexin	68	*CID 5280441*	*M. chamissois*	Leaves	Antimicrobial, cytotoxic and Antioxidant	[6]
**Phenolics**						
1,2,3,5-tetra-galloy l-4,6-HHDPglucose	69	-	*M. latecrenata*	Leaves	Antioxidant, antibacterial and antimutagenic	[16]
					Antiplasmodial	[16]
1-galloyl-2,3; 4,6-bis-HHDPglucose isomer	70	-	*M. latecrenata*	Leaves	Antibacterial	[47]
					Antioxidant Antimutagenic	[16]
1-*O*-(*E*)-caffeoyl-4,6-di-*O*-galloyl-β-*D*-glucopyranose	71	-	*M. albicans*	Leaves	Antidiabetic	[65]
2-Cinnamoyl-1-galloy-l-β-Dglucopyranose	72	*CID 131752569*	*M. albicans*	Fruits	Antioxidant and anti-inflammatory	[13]
2,3; 4,6-bis-HHDP-glucose isomer	73	-	*M. latecrenata*	Leaves	Antibacterial	[47]
					Antioxidant and antimutagenic	[16]

					Antiplasmodial	[16]
3,3′-di-O-methyl ellagic acid-4-O-β-*D*-xylopyranoside	74	-	*M. myriantha*	Aerial parts	-	[67]
3,3′,4-Tri-O-methylellagic acid	75	-	*M. albicans*	Fruits	Antioxidant and anti-inflammatory	[13]
3,3′-di-O-methyl elagic acid	76	*CID 5488919*	*M. albicans*	Fruits	Antioxidant and anti-inflammatory	[13]
3,4-Dihydroxybenzoic acid	77	*CID 72*	*M. albicans*	Fruits	Antioxidant and anti-inflammatory	[13]
4-Hydroxybenzoate-O-glucoside	78	-	*M. albicans*	Fruits	Antioxidant and anti-inflammatory	[13]
4-Hydroxybenzoic acid	79	*CID 135*	*M. albicans*	Fruits	Antioxidant and anti-inflammatory	[13]
Caffeic acid	80	*CID 689043*	*M. chamissois*	Leaves	Antimicrobial, cytotoxic and Antioxidant	[6]
Casuarictin	81	*CID 73644*	*M. rubiginosa*	Leaves	-	[61]
Casuarinin isomer	82	*CID 442673*	*M. minutiflora*	Leaves	Anti-inflammatory and antinociceptive	[14]
Cinnamic acid	83	*CID 444539*	*M. albicans*	Fruits	Antioxidant and anti-inflammatory	[13]
Ellagic acid pentoside	84	-	*M. latecrenata*	Leaves	Antioxidant Antibacterial and antimutagenic	[16]
Ellagic acid rhamnoside	85	-	*M. latecrenata*	Leaves	Antioxidant, antibacterial and antimutagenic	[16]
Ellagic acid	86	*CID 5281855*	*M. myriantha*	Aerial parts	-	[67]
			M. minutiflora	Leaves	Anti-inflammatory and antinociceptive	[14]
			M. latecrenata		Antibacterial	[47]
Ethyl gallate	87	*CID 553710*	*M. albicans*	Leaves	Anti-inflammatory	[32]
Gallic acid	88	*CID 370*	*M. rubiginosa*	Leaves	-	[61]
			M. albicans		Anti-inflammatory	[32]
				Fruits	Antioxidant and anti-inflammatory	[13]
			M. myriantha	Aerial parts	-	[67]
			M. cabucu	Aerial parts	-	[62]
HHDP galloylglucose	89	-	*M. minutiflora*	Leaves	Anti-inflammatory and antinociceptive	[14]
HHDP-Hexoside	90	-	*M. albicans*	Leaves	Anti-inflammatory	[32]
Malic acid	91	*CID 525*	*M. albicans*	Fruits	Antioxidant and anti-inflammatory	[13]
Methyl gallate	92	*CID 7428*	*M. albicans*	Fruits	Antioxidant and anti-inflammatory	[13]
Miconidin	93	*CID 169581*	*M. willdenowii*	Leaves	Leishmanicidal and antimicrobial	[18]
p-Coumaric acid	94	*CID 637542*	*M. albicans*	Fruits	Antioxidant and anti-inflammatory	[13]
Pedunculagin	95	*CID 442688*	*M. albicans*	Leaves	Anti-inflammatory	[32]
Pyrocatechol	96	*CID 66993*	*M. albicans*	Fruits	Antioxidant and anti-inflammatory	[13]

Quinic acid	97	*CID 6508*	*M. albicans*	Fruits	Antioxidant and anti-inflammatory	[13]
Salicylic acid	98	*CID 338*	*M. albicans*	Fruits	Antioxidant and anti-inflammatory	[13]
Schizandriside	99	*CID 14521043*	*M. rubiginosa*	Leaves	-	[61]
Tris-galloyl-HHDP-glucose isomer	100	-	*M. latecrenata*	Leaves	Antioxidant, antibacterial and antimutagenic	[16]
					Antiplasmodial	[16]
β-Glucogallin	101	*CID 124021*	*M. albicans*	Fruits	Antioxidant and anti-inflammatory	[13]
**Terpenoids**						
(+)-Trans-carveol	102	*CID 94221*	*M. albicans*	Fruits	Antioxidant and anti-inflammatory	[13]
1-Octen-3-ol	103	*CID 18827*	*M. ferruginata*	Aerial parts	-	[68]
Eugenol	104	*CID 3314*	*M. ferruginata*	Aerial parts	-	[68]
Germacrene D	105	*CID 5317570*	*M. ferruginata*	Aerial parts	-	[68]
L-Borneol	106	*CID 1201518*	*M. ferruginata*	Aerial parts	-	[68]
α-Copaene	107	*CID 70678558*	*M. ferruginata*	Aerial parts	-	[68]
α-Humulene	108	*CID 24798693*	*M. ferruginata*	Aerial parts	-	[68]
β-Caryophyllene	109	*CID 5281515*	*M. ferruginata*	Aerial parts	-	[68]
β-Cubebene	110	*CID 93081*	*M. ferruginata*	Aerial parts	-	[68]
β-Elemene	111	*CID 6918391*	*M. ferruginata*	Aerial parts	-	[68]
**Triterpenes and steroids**						
28-carboxy-3-oxoolean-12-en-21a-yl acetate	112	-	*M. macrothyrsa*	Leaves	-	[69]
2α- hydroxyursolic acid	113	-	*M. ligustroides* *M. sellowiana*	Aerial parts	Antiparasitic	[34]
			M. sellowiana		Antibacterial	[41]
2α,3α,19α, 23-tetrahydroxyurs-12-ene-28-oic acid (myrianthic acid)	114	*CID 182497*	*M. trailii*	Aerial parts	-	[64]
2α,3β,19α-trihydroxyolean-12-ene-24,28-dioic acid (bartogenic acid)	115	*CID 45272347*	*M. trailii*	Aerial parts	-	[64]
3-(*E*)-*p*-coumaroyl-α-amyrin	116	-	*M. albicans*	Leaves	Antioxidant	[51]
3-*epi*-sumaresinolic acid	117	-	*M. albicans*	Leaves	Antidiabetic	[65]
3-*O*-*cis*-*p*-coumaroyl maslinic acid	118	-	*M. albicans*	Leaves	Antidiabetic	[65]
3-*O*-*trans*-*p*-coumaroyl 2α-hydroxydulcioic acid	119	-	*M. albicans*	Leaves	Antidiabetic	[65]
3-*O*-*trans*-*p*-coumaroyl maslinic acid	120	CID 16664517	*M. albicans*	Leaves	Antidiabetic	[65]
Arjunolic acid	121	*CID 73641*	*M. trailii*	Aerial parts	-	[64]
			M. ligustroides *M. sellowiana*	Aerial parts	Antiparasitic	[33]
			M. minutiflora	Leaves	Anti-inflammatory and antinociceptive	[14]
			M. albicans	Fruits	Antioxidant and anti-inflammatory	[13]

Asiatic acid	122	*CID 119034*	*M. albicans*	Fruits	Antioxidant and anti-inflammatory	[13]
Campesterol	123	*CID 173183*	*M. sellowiana*<br>*M. albicans*<br>*M. pepericarpa*	Aerial parts	-	[34]
epi-Betulinic acid	124	*CID 485711*	*M. albicans*	Leaves	Antioxidant	[51]
epi-ursolic acid	125	-	*M. albicans*	Leaves	Antioxidant	[51]
Friedelin	126	*CID 91472*	*M. pepericarpa*	Aerial parts	-	[34]
Gypsogenic acid	127	*CID 15560324*	*M. fallax*<br>*M. stenostachya*	Aerial parts	Trypanocide	[33]
			M. stenostachya		Antibacterial	[41]
Lupeol	128	*CID 259846*	*M. rubiginosa*<br>*M. fallax*<br>*M. sellowiana*<br>*M. albicans*<br>*M. pepericarpa*	Aerial parts	-	[34]
			M. burchellii.	Leaves	Cytotoxic	[55]
Maslinic acid	129	*CID 73659*	*M. ligustroides*<br>*M. sellowiana*	Aerial parts	Antiparasitic	[34]
			M. stenostachya		Antibacterial	[41]
			M. albicans	Leaves	Antidiabetic	[65]
				Fruits	Antioxidant and anti-inflammatory	[13]
Myrianthic acid isomer	130	*CID 182497*	*M. minutiflora*	Leaves	Anti-inflammatory and antinociceptive	[14]
			M. albicans	Fruits	Antioxidant and anti-inflammatory	[13]
Oleanolic acid	131	*CID 10494*	*M. fallax*<br>*M. stenostachya*	Aerial parts	Trypanocide	[33]
			M. ligustroides<br>*M. sellowiana*		Antiparasitic	[34]
			M. langsdorffii		Antileishmanial	[28]
			M. fallax		Antibacterial and antitumor	[41,57]
			M. albicans		Analgesic and anti-inflammatory	[15]
					Antidiabetic	[65]
			M. rubiginosa		-	[19]
			M. albicans	Fruits	Antioxidant and anti-inflammatory	[13]
			M. ligustroides	Leaves	Antibacterial	[44]
			M. ferruginata		Insecticide	[26]
			M. chamissois			[66]
			M. burchellii.	Leaves	Cytotoxic	[55]
Stigmast-4-ene-3,6-dione	132	*CID 5490007*	*M. trailii*	Aerial parts	-	[64]
Stigmasterol	133	*CID 5280794*	*M. fallax*<br>*M. sellowiana*<br>*M. ligustroides*<br>*M. albicans*<br>*M. pepericarpa*	Aerial parts	-	[34]
			M. ferruginata	Leaves	Insecticide	[26]
Sumaresinolic acid	134	*CID 4660579*	*M. fallax*<br>*M. stenostachya*	Aerial parts	Antibacterial	[41]

			M. albicans	Leaves	Antidiabetic	[65]
Ursolic acid	135	*CID 13073356*	*M. ligustroides*<br>*M. sellowiana*	Aerial parts	Antiparasitic	[34]
			M. fallax<br>*M. stenostachya*		Trypanocide	[33]
			M. fallax<br>*M. albicans*		Antibacterial<br>Antitumor	[41]<br>[57]
					Analgesic and anti-inflammatory	[15]
					Antidiabetic	[65]
			M. langsdorffii		Antileishmanial	[28]
			M. rubiginosa			[19]
					Antioxidant	[51]
			M. ligustroides	Leaves	Antibacterial	[44]
			M. ferruginata		Insecticide	[26]
			M. chamissois			[66]
			M. burchellii.		Cytotoxic	[55]
α-Amyrin acetate	136	*CID 293754*	*M. pepericarpa*	Aerial parts	-	[34]
α-Amyrin	137	*CID 73170*	*M. rubiginosa*<br>*M. fallax*<br>*M. sellowiana*<br>*M. ligustroides*<br>*M. pepericarpa*	Aerial parts	-	[34]
			M. albicans	Leaves	Antioxidant	[51]
β-Amyrin acetate	138	*CID 92156*	*M. pepericarpa*	Aerial parts	-	[34]
β-Amyrin	139	*CID 73145*	*M. rubiginosa*<br>*M. fallax*<br>*M. sellowiana*<br>*M. ligustroides*<br>*M. albicans*<br>*M. pepericarpa*	Aerial parts	-	[34]
β-Sitosterol	140	*CID 222284*	*M. rubiginosa*<br>*M. fallax*<br>*M. sellowiana*<br>*M. ligustroides*<br>*M. albicans*<br>*M. pepericarpa*	Aerial parts	-	[34]
			M. ferruginata	Leaves	Insecticide	[26]
			M. burchellii.	Leaves	Cytotoxic	[55]
**Others compounds**						
2-methoxy-6-heptyl-1,4-benzoquinone	141	-	*M. lepidota*	Leaves	Antimicrobial and cytotoxic	[38]
8-Heptadecene	142	*CID 5364555*	*M. ferruginata*	Aerial parts	-	[68]
8-Hexadecyne	143	*CID 123387*	*M. ferruginata*	Aerial parts	-	[68]
9,10-Dihydroxy-8-oxooctadec-12-enoic acid	144	*CID 71342303*	*M. albicans*	Fruits	Antioxidant and anti-inflammatory	[13]
Ethyl ester	145	*CID 5282208*	*M. burchellii.*	Leaves	Cytotoxic	[55]
Hexadecane	146	*CID 11006*	*M. ferruginata*	Aerial parts	-	[68]
Pentadecane	147	*CID 12391*	*M. ferruginata*	Aerial parts	-	[68]

Primin	148	*CID 84800*	*M. willdenowii*	Leaves	Schistosomicidal	[17]
					Leishmanicidal and antimicrobial	[18]
			M. lepidota		Antimicrobial and cytotoxic	[38]

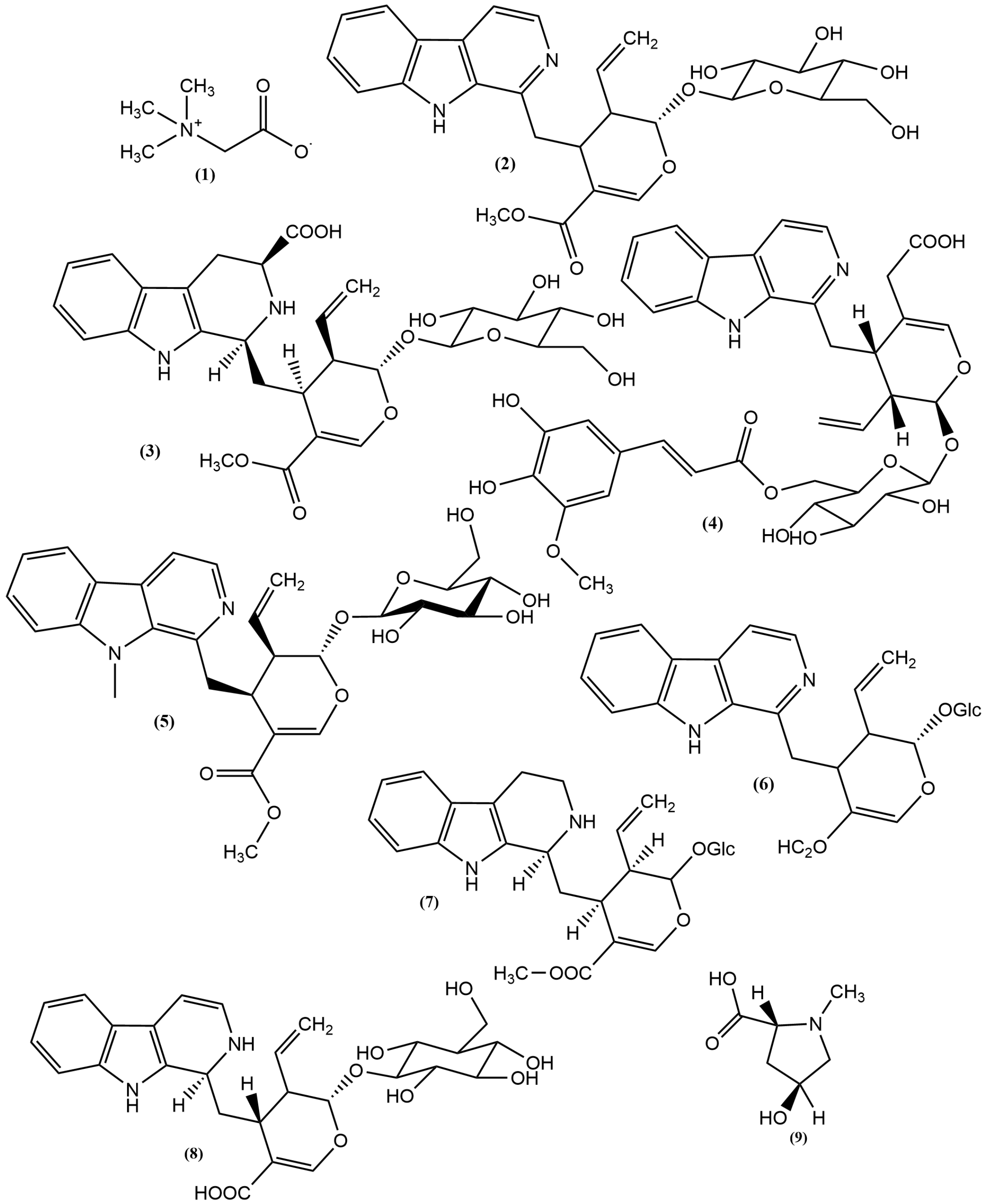

Figure S1. Alkaloids identified in the species of the genus *Miconia* (Compounds 1-9).

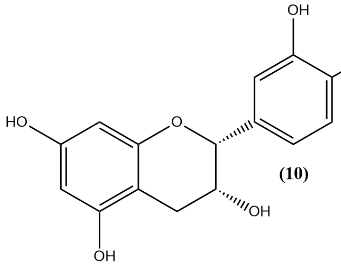
(10)

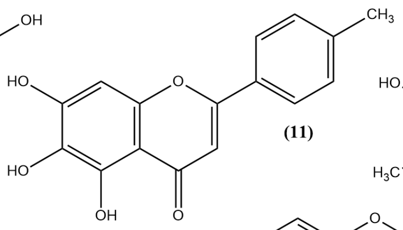
(11)

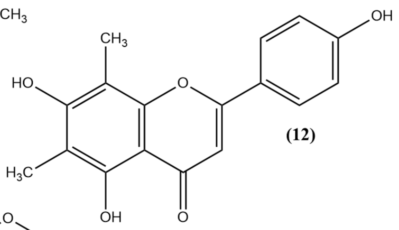
(12)

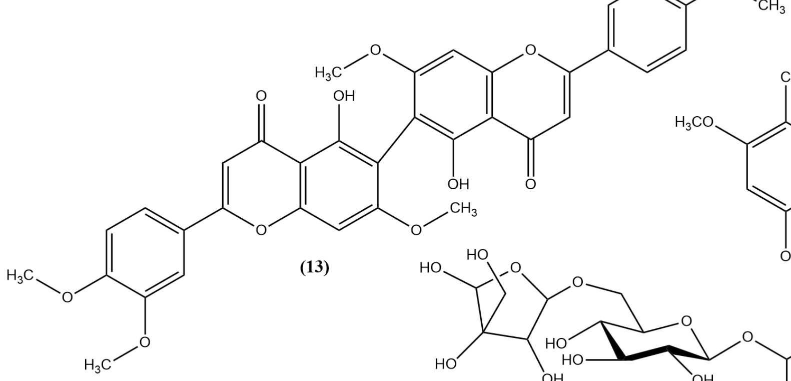
(13)

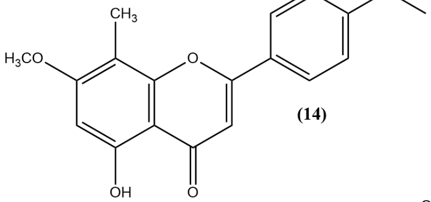
(14)

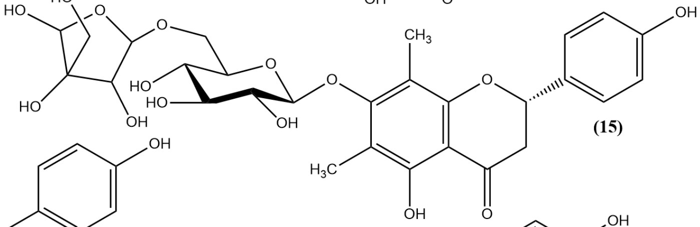
(15)

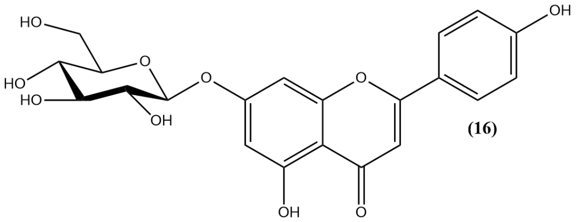
(16)

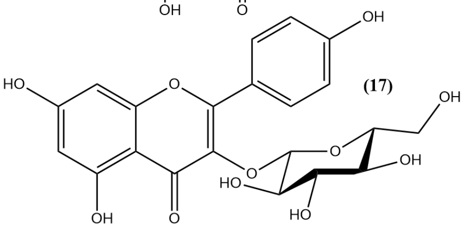
(17)

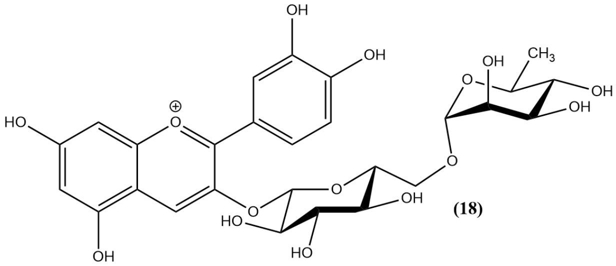
(18)

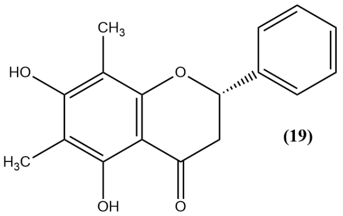
(19)

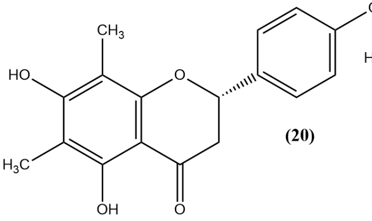
(20)

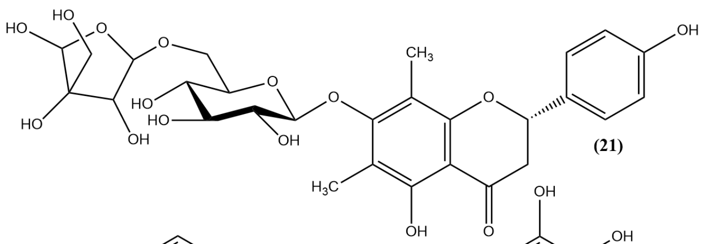
(21)

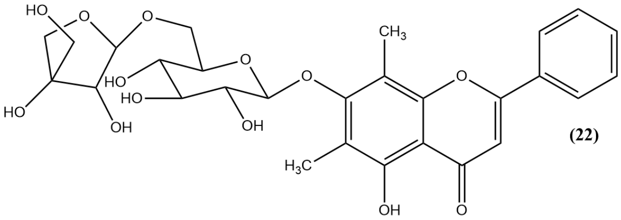
(22)

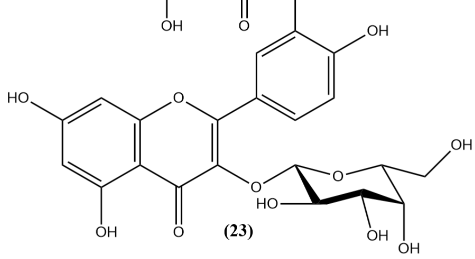
(23)

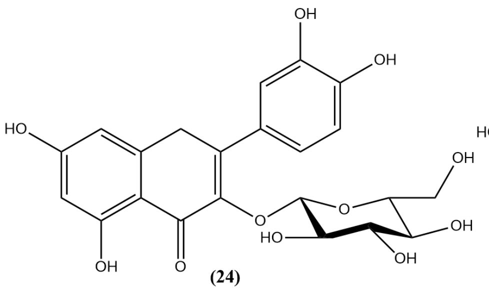

(24)

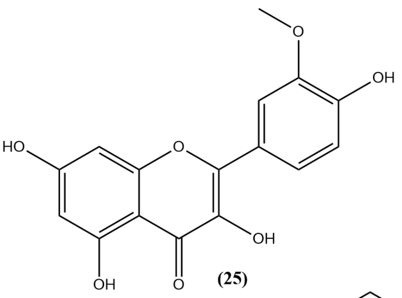

(25)

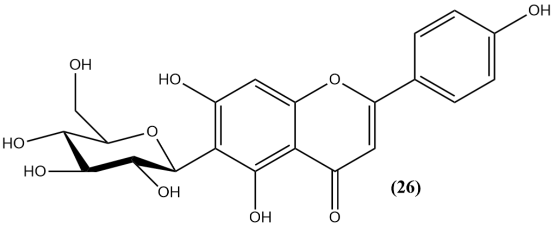

(26)

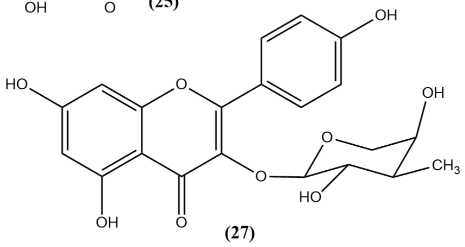

(27)

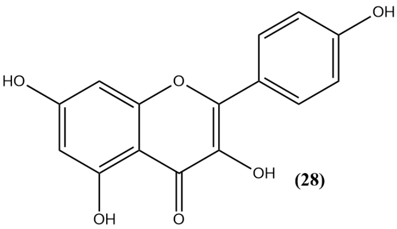

(28)

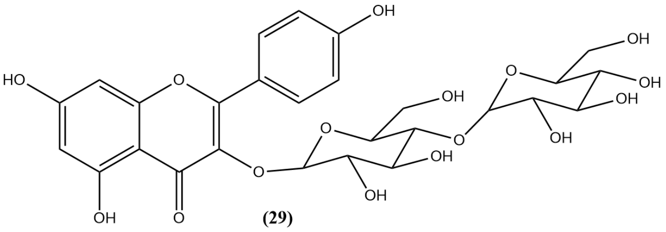

(29)

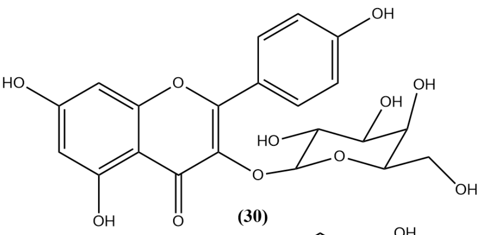

(30)

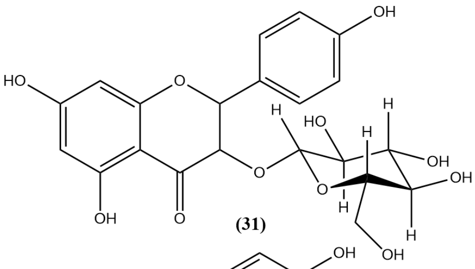

(31)

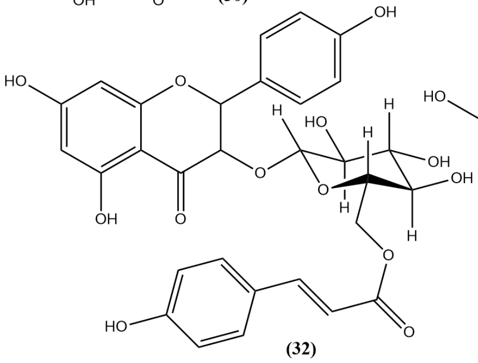

(32)

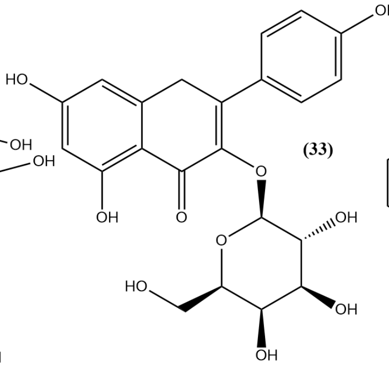

(33)

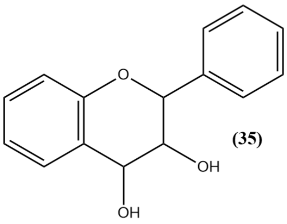

(35)

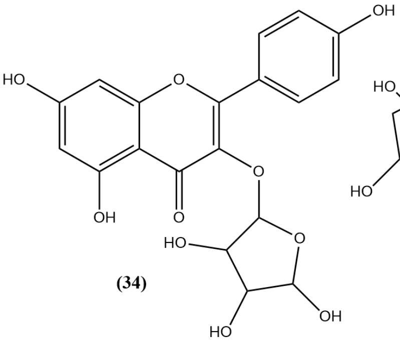

(34)

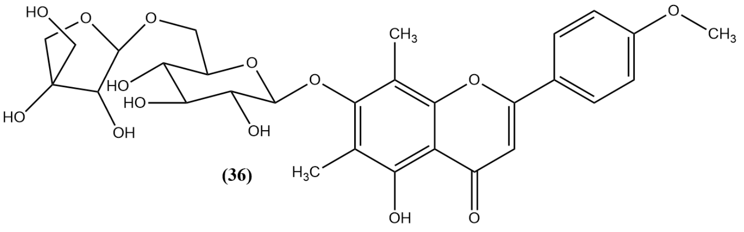

(36)

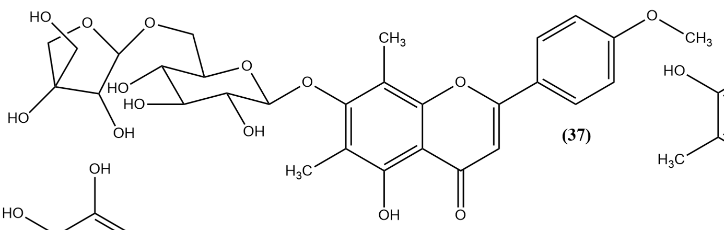

(37)

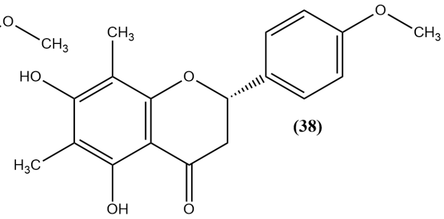

(38)

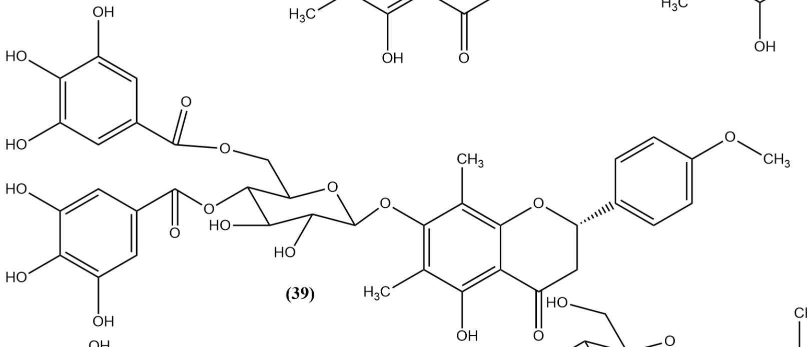

(39)

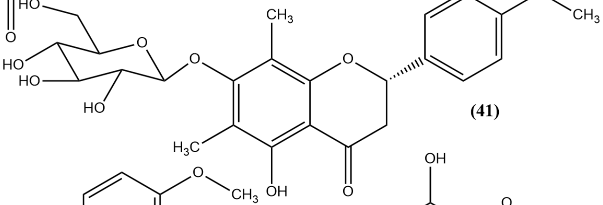

(41)

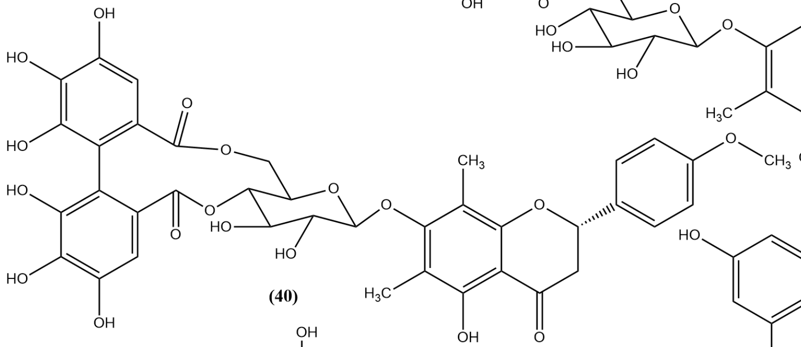

(40)

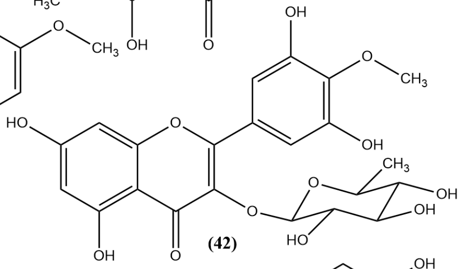

(42)

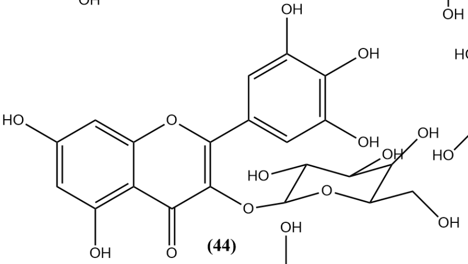

(44)

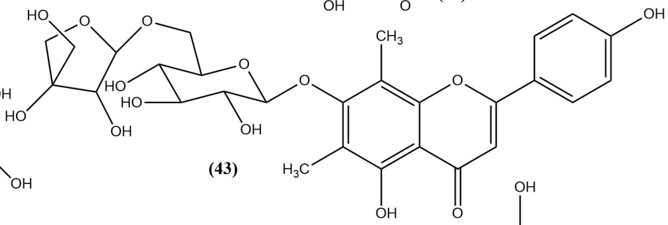

(43)

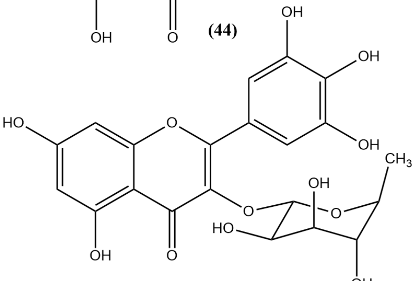

(45)

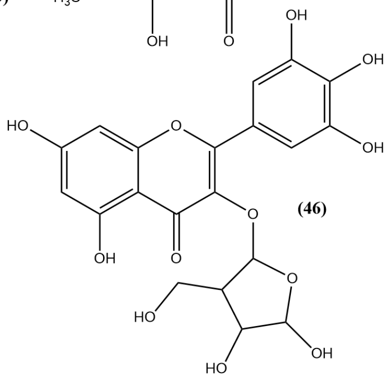

(46)

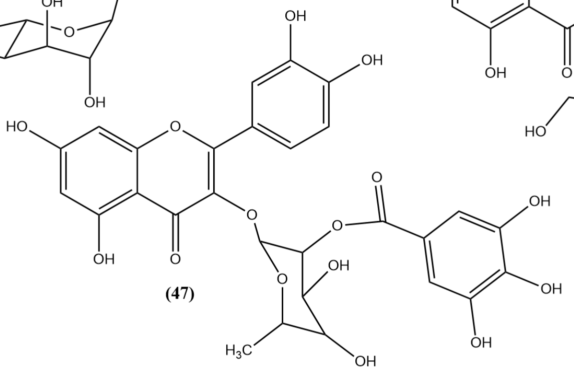

(47)

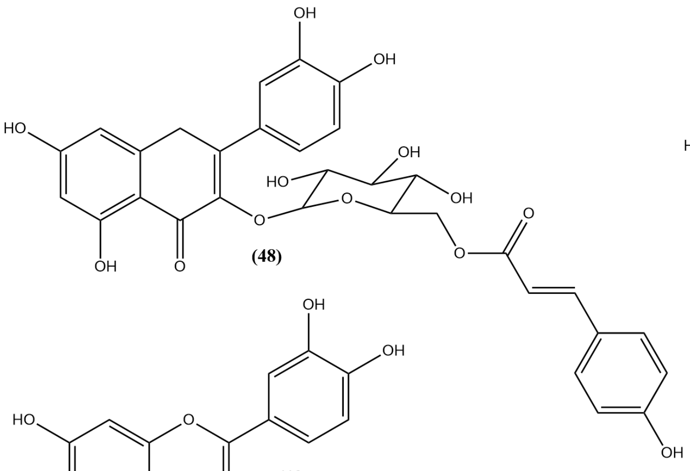

(48)

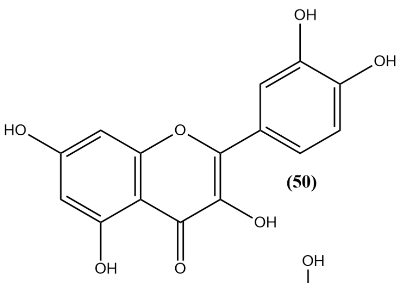

(50)

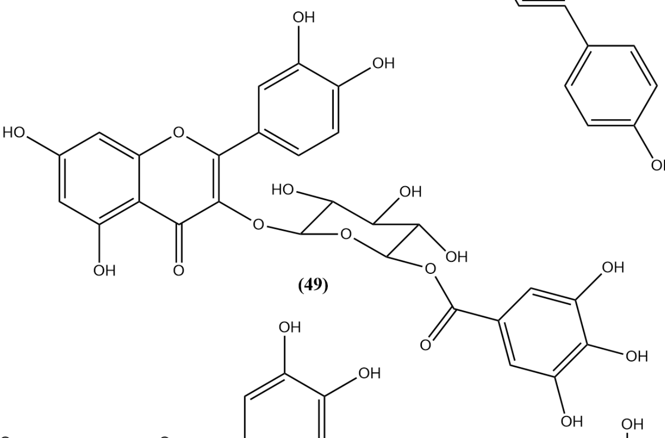

(49)

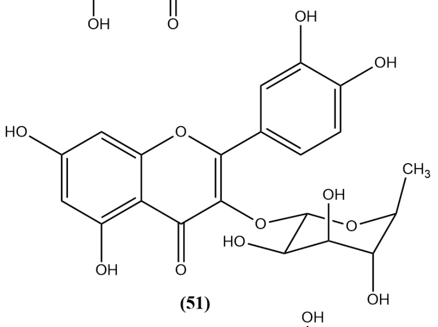

(51)

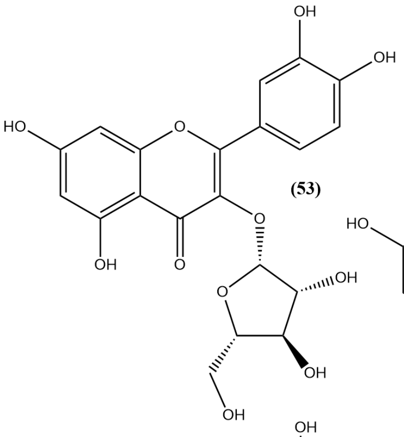

(53)

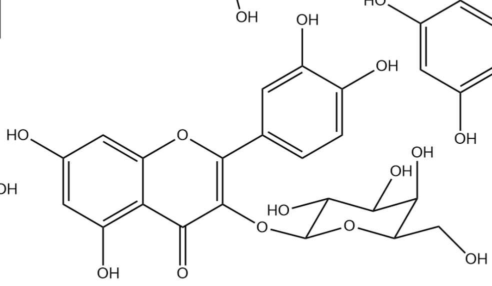

(54)

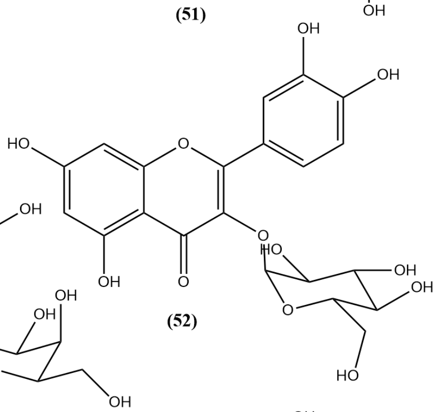

(52)

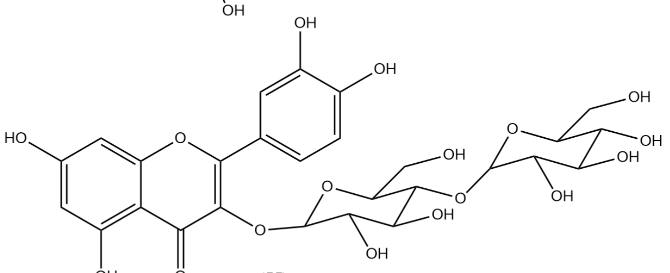

(55)

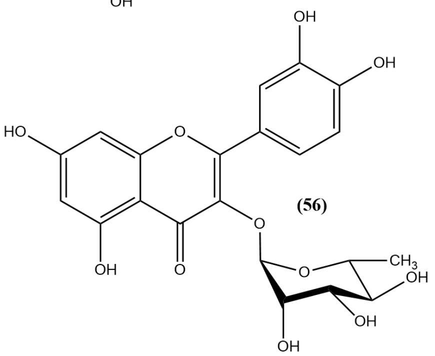

(56)

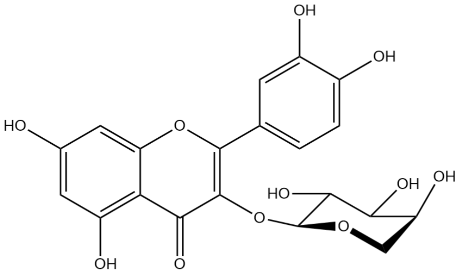

(57)

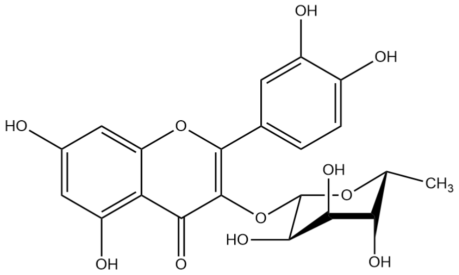

(58)

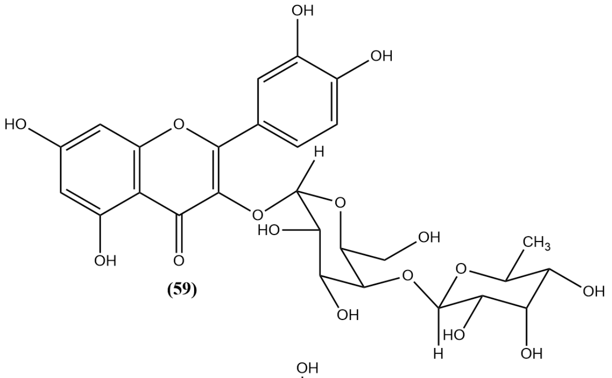

(59)

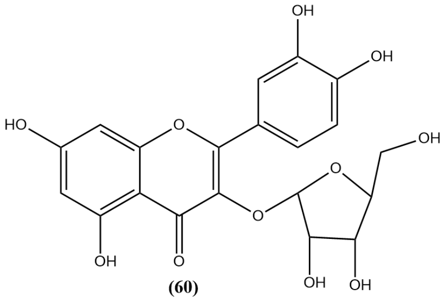

(60)

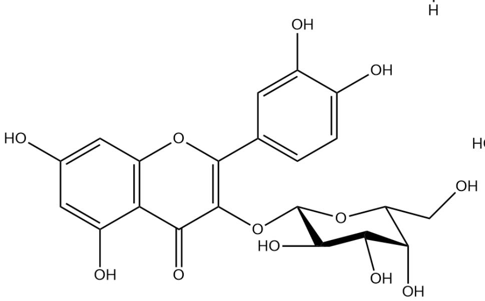

(61)

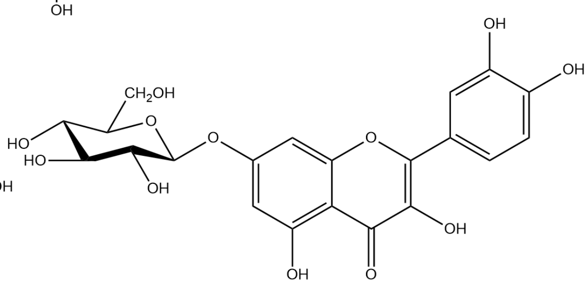

(62)

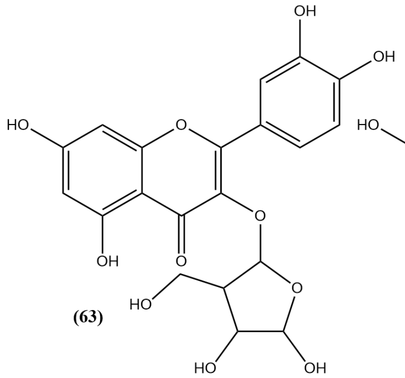

(63)

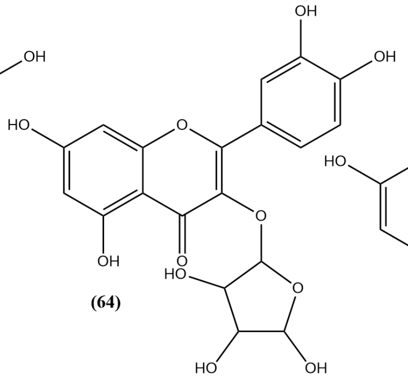

(64)

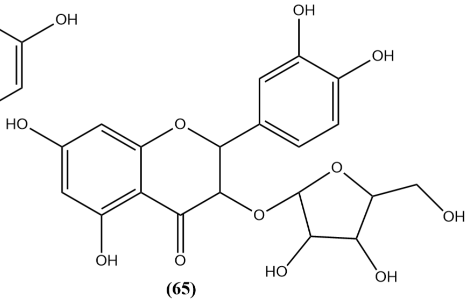

(65)

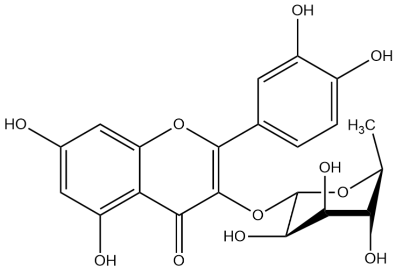

(66)

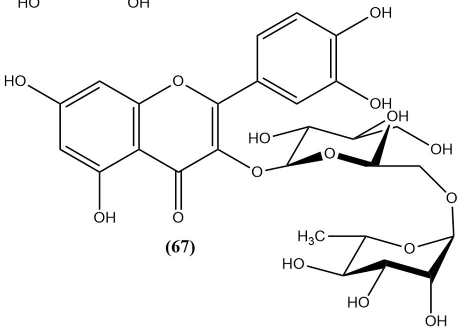

(67)

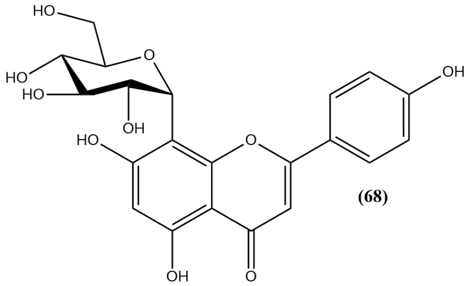

(68)

Figure S2. Flavonoids identified in the species of the genus *Miconia* (Compounds 10-68).

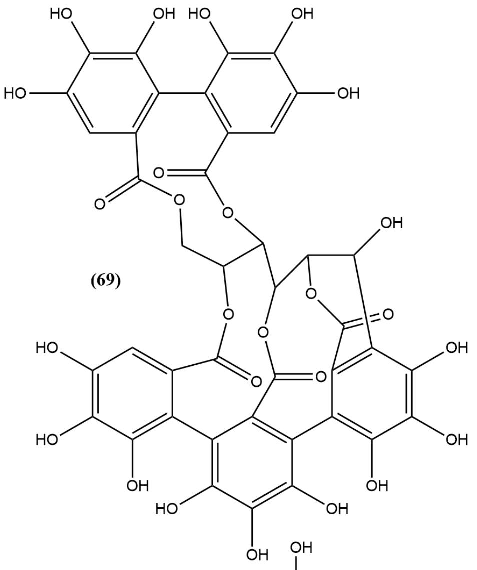

(69)

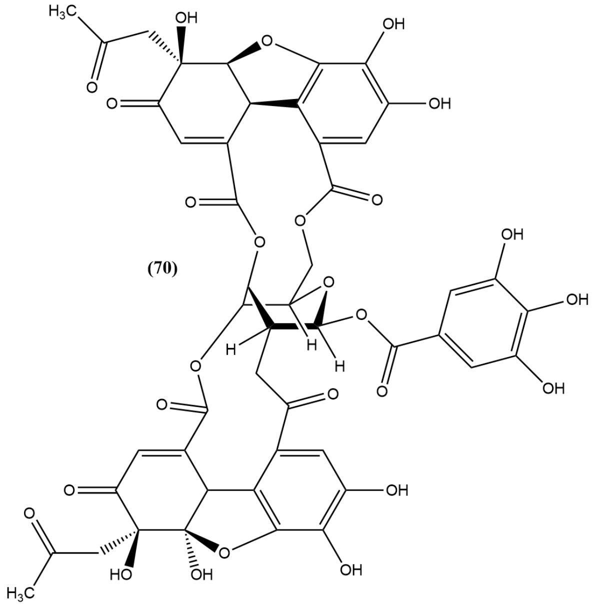

(70)

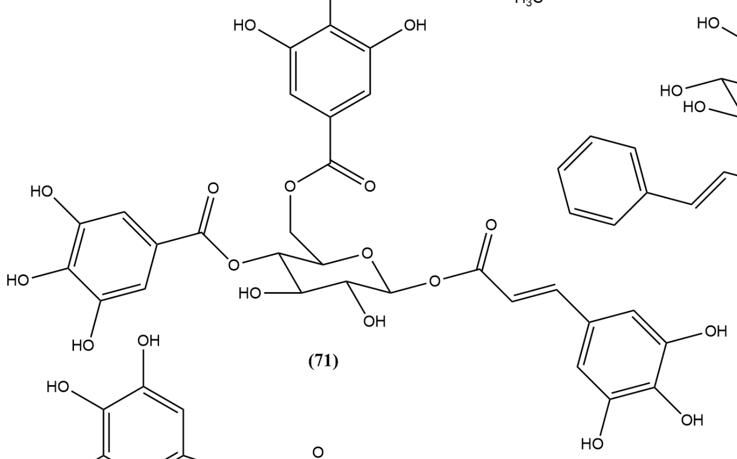

(71)

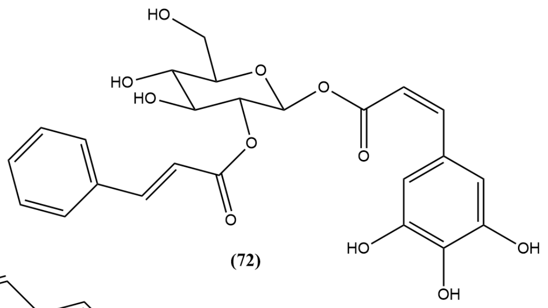

(72)

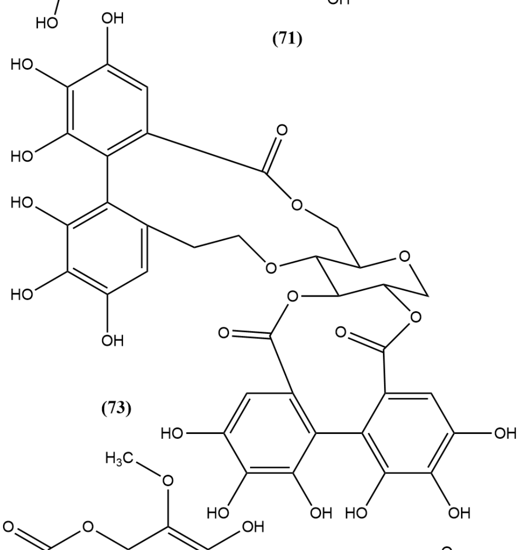

(73)

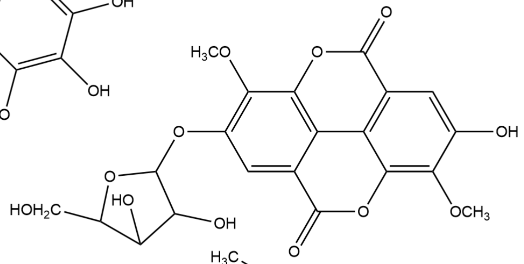

(74)

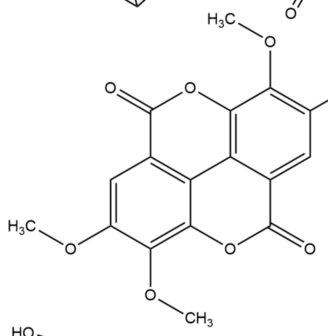

(75)

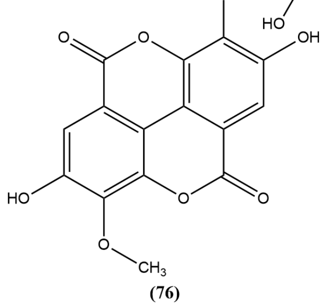

(76)

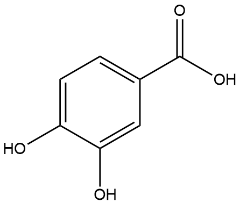

(77)

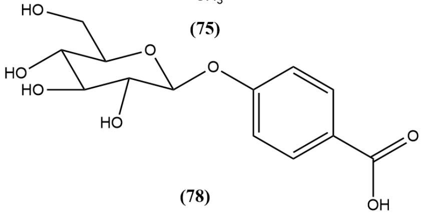

(78)

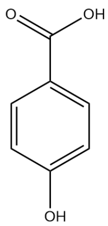

(79)

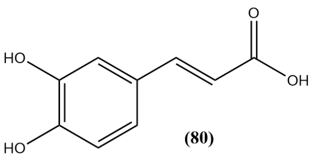

(80)

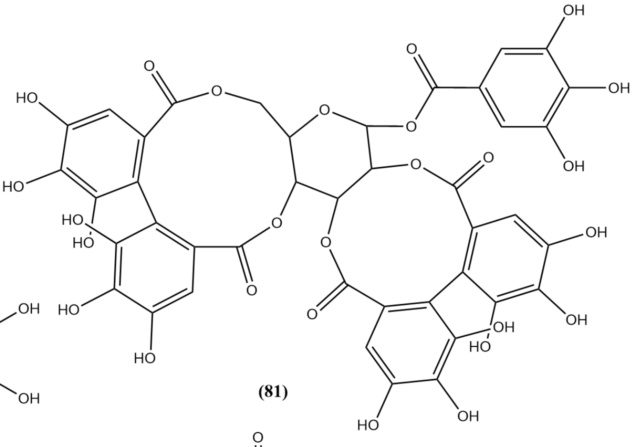

(81)

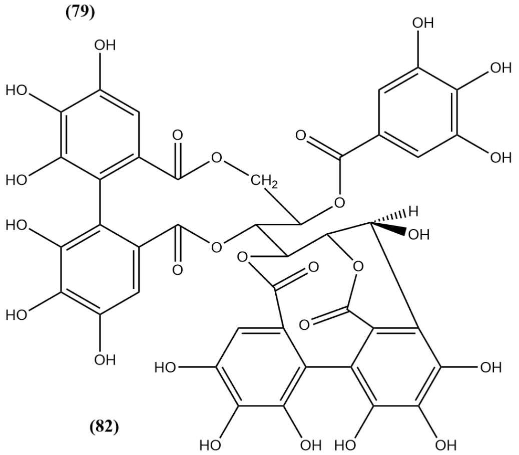

(82)

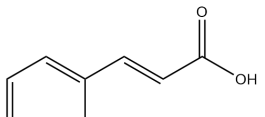

(83)

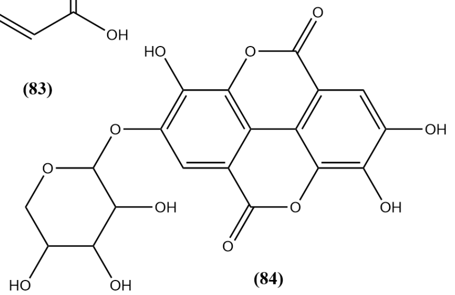

(84)

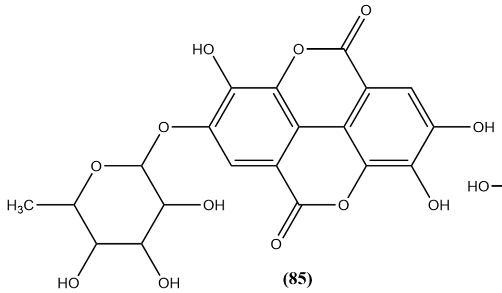

(85)

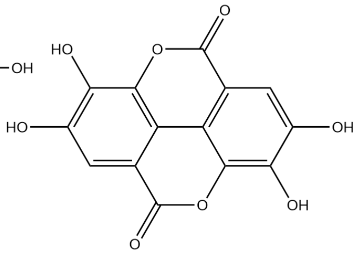

(86)

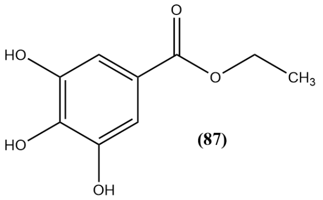

(87)

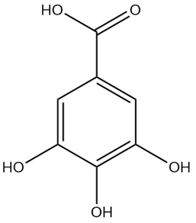

(88)

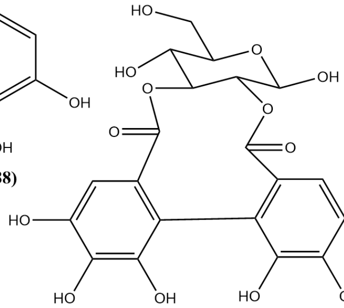

(90)

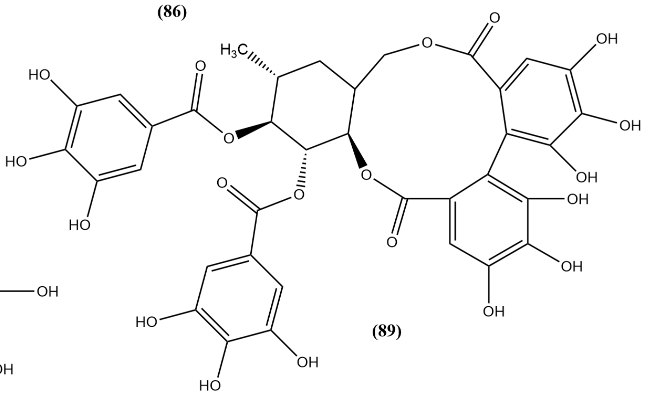

(89)

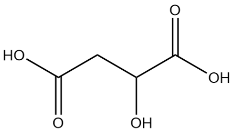

(91)

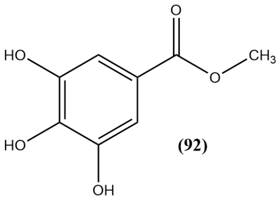

(92)

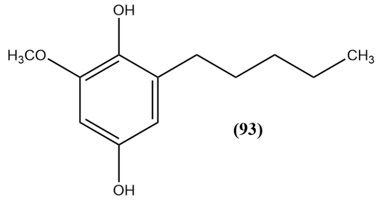

(93)

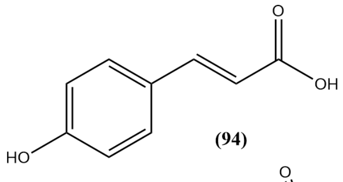

(94)

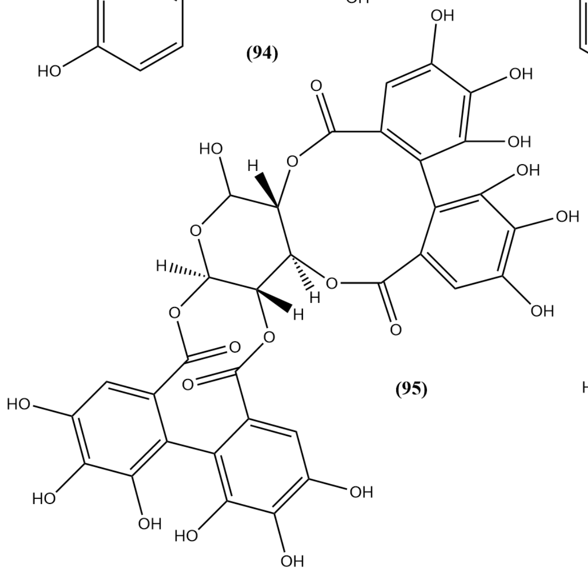

(95)

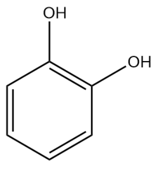

(96)

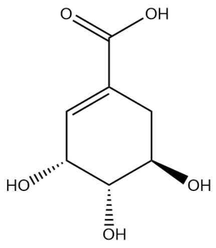

(97)

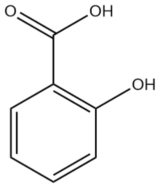

(98)

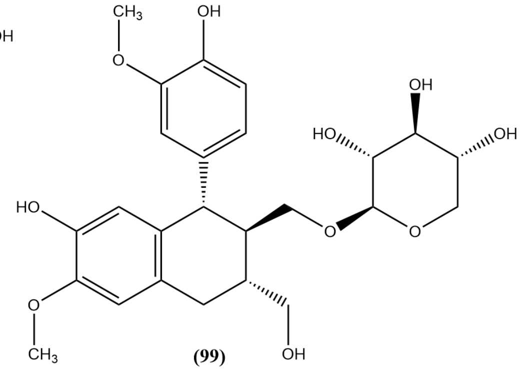

(99)

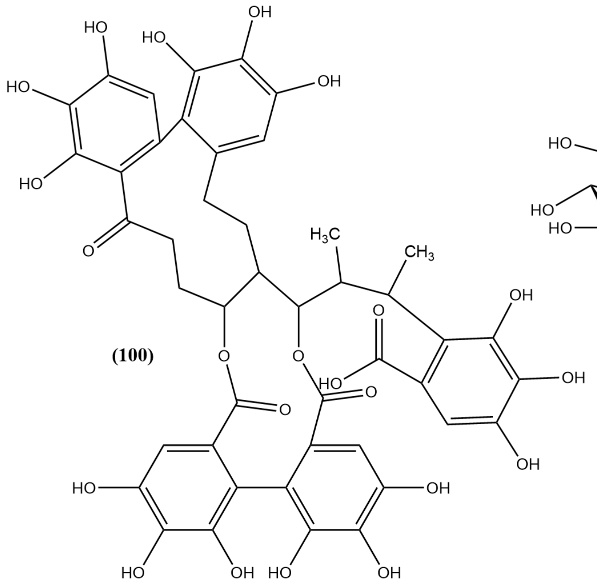

(100)

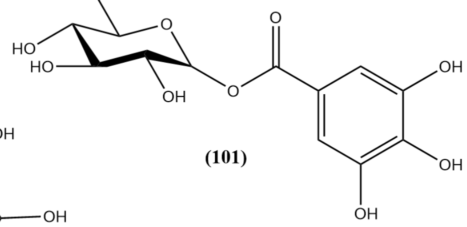

(101)

Figure S3. Phenolics identified in the species of the genus *Miconia* (Compounds 69-101).

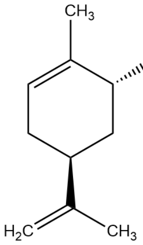

(102)

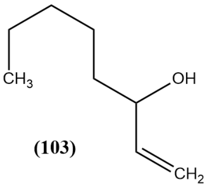

(103)

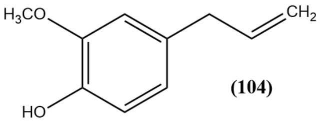

(104)

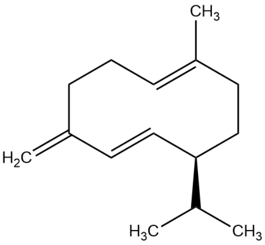

(105)

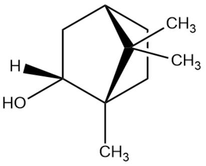

(106)

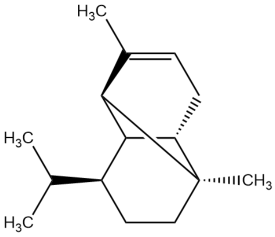

(107)

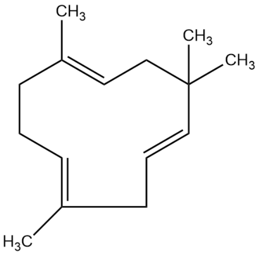

(108)

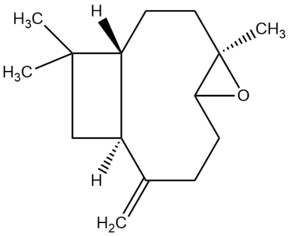

(109)

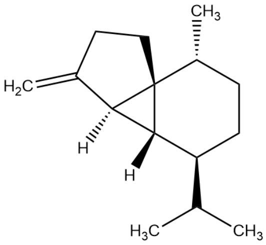

(110)

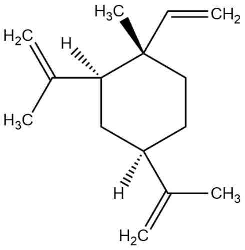

(111)

Figure S4. Terpenoids identified in the species of the genus *Miconia* (Compounds 102-111).

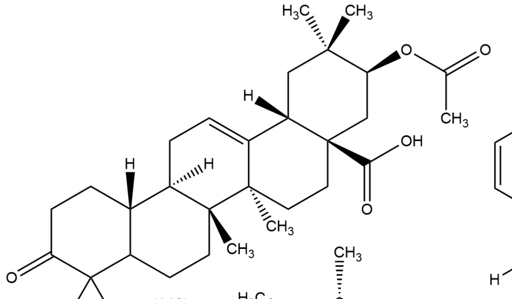

(112)

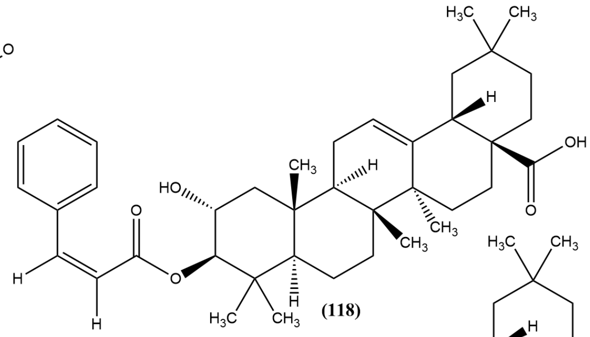

(118)

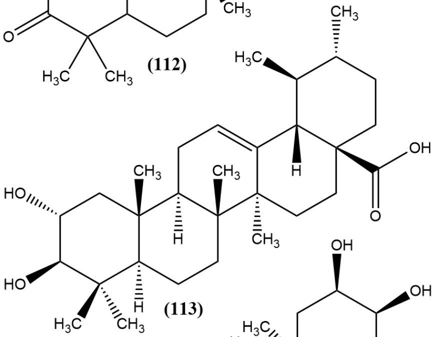

(113)

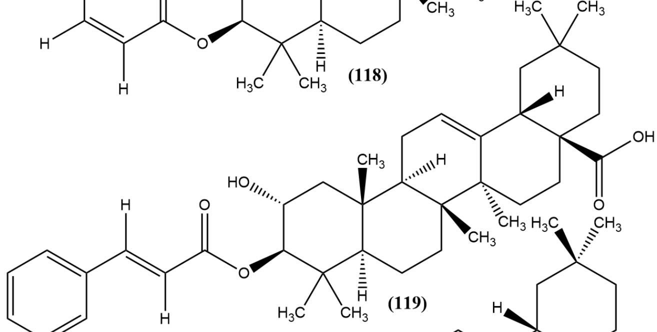

(119)

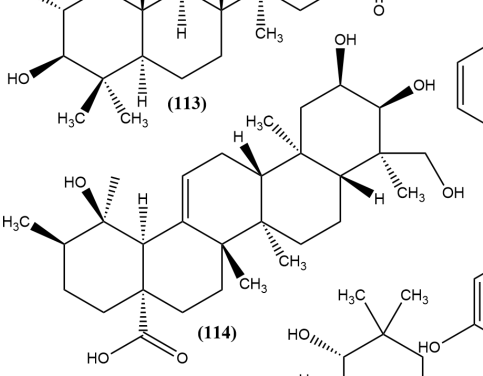

(114)

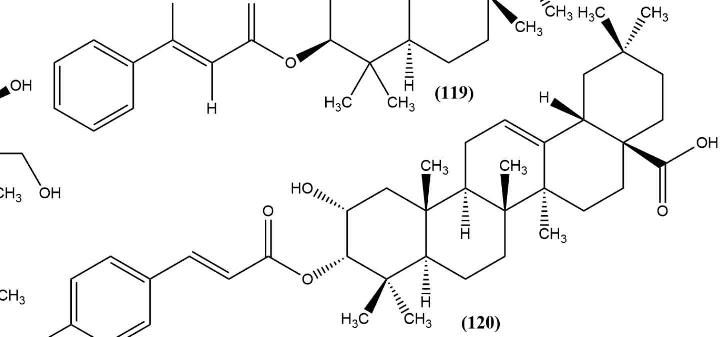

(120)

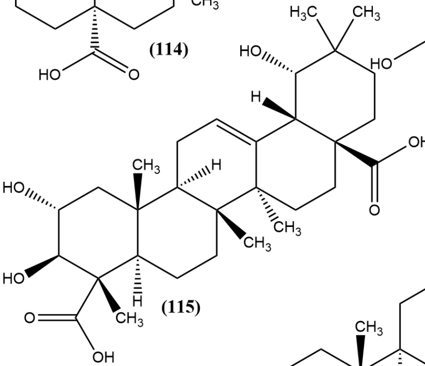

(115)

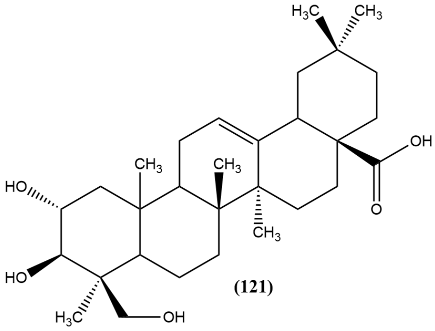

(121)

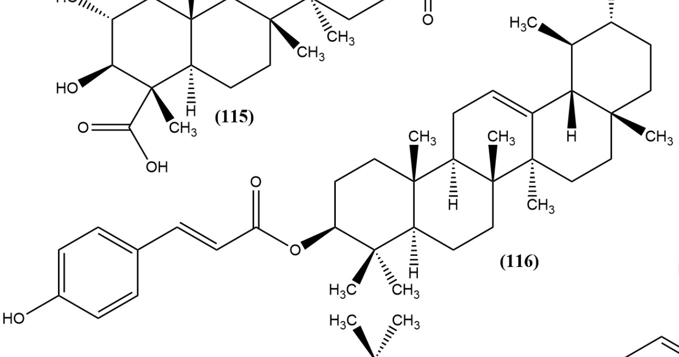

(116)

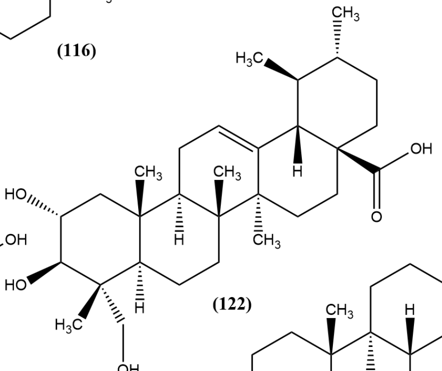

(122)

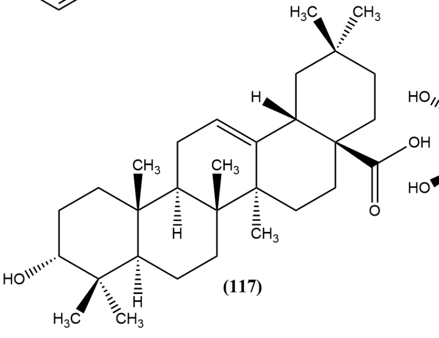

(117)

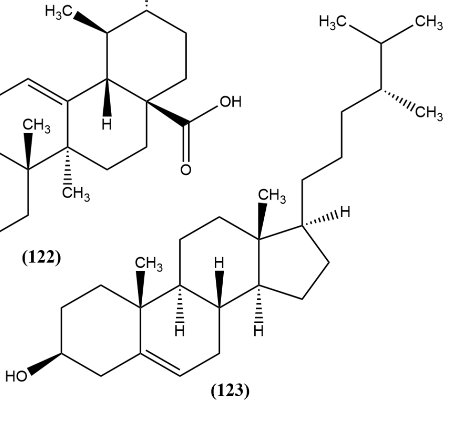

(123)

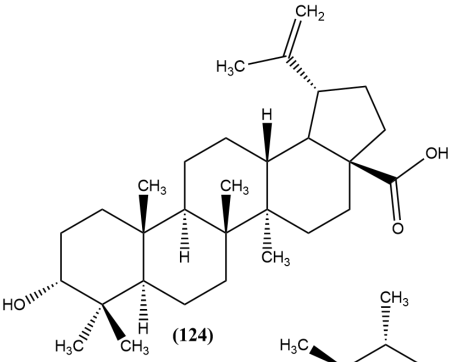
(124)

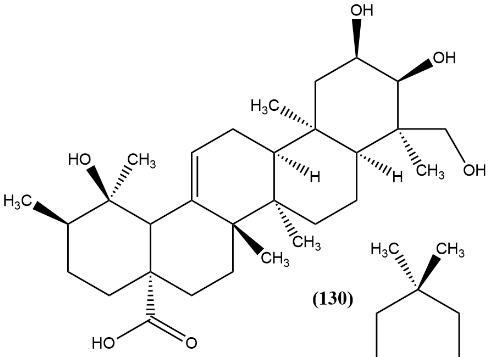
(130)

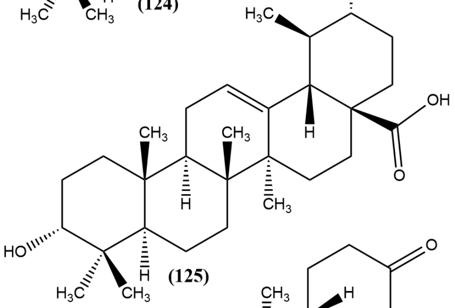
(125)

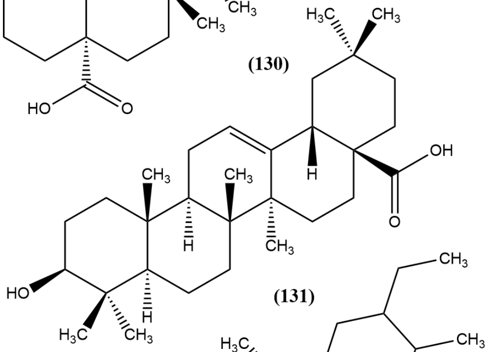
(131)

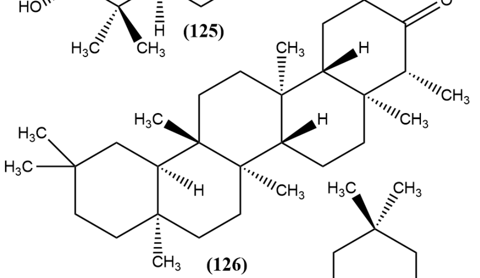
(126)

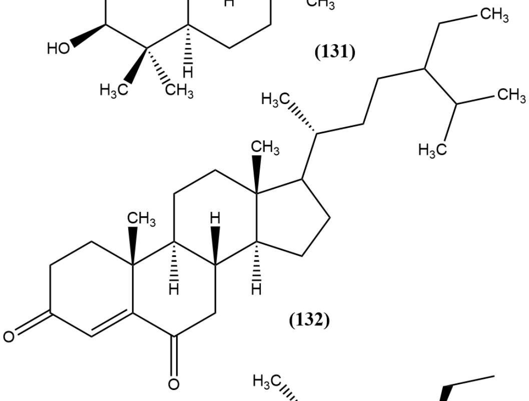
(132)

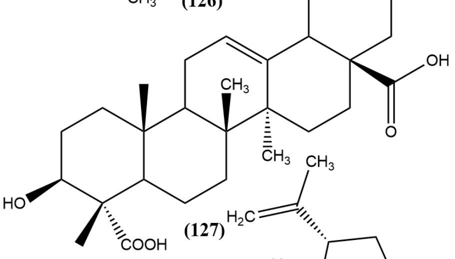
(127)

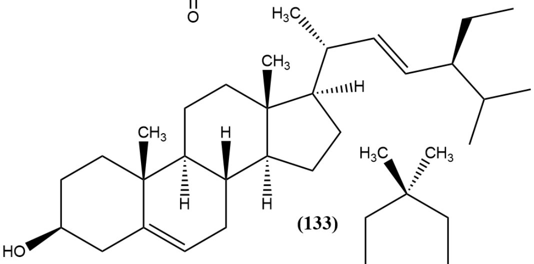
(133)

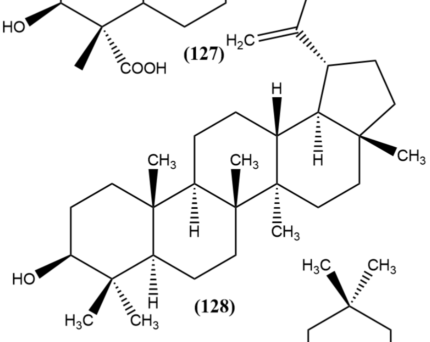
(128)

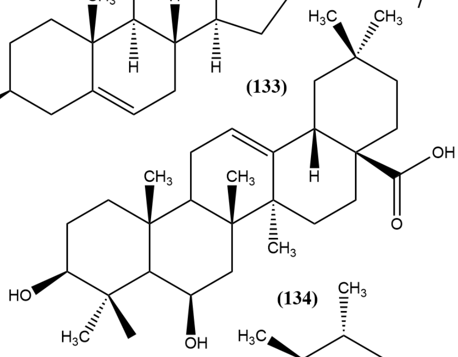
(134)

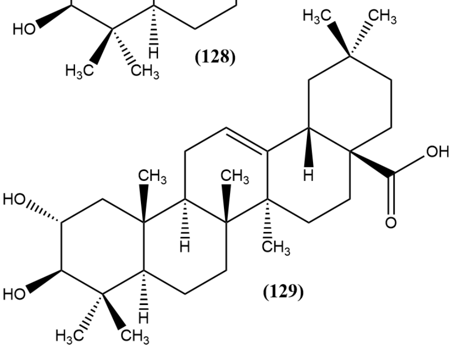
(129)

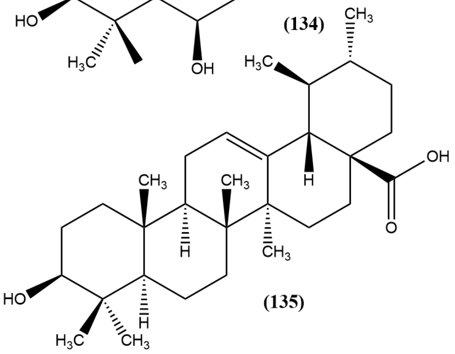
(135)

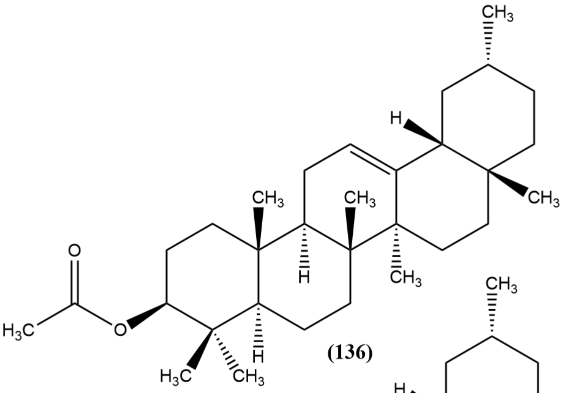

(136)

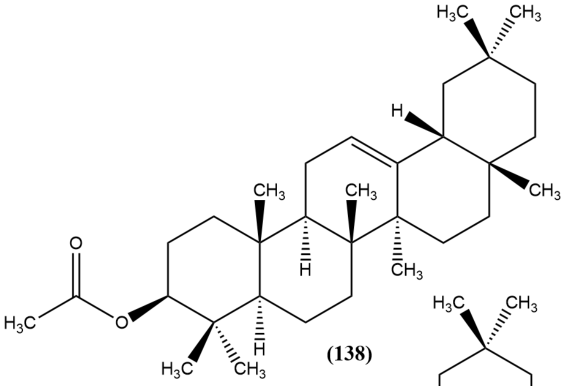

(138)

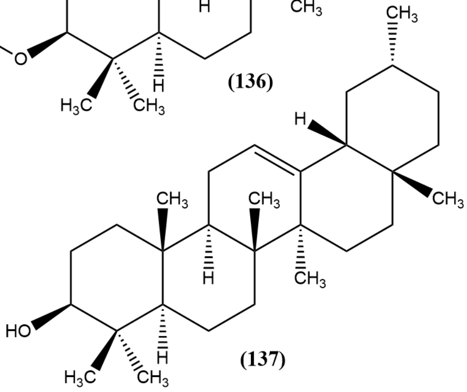

(137)

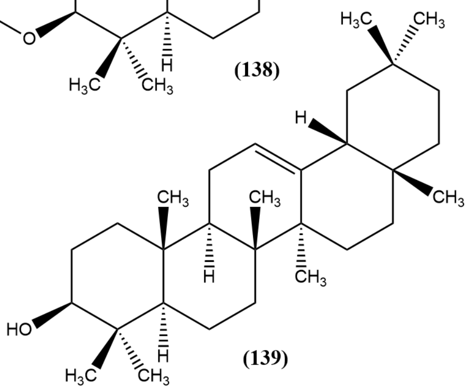

(139)

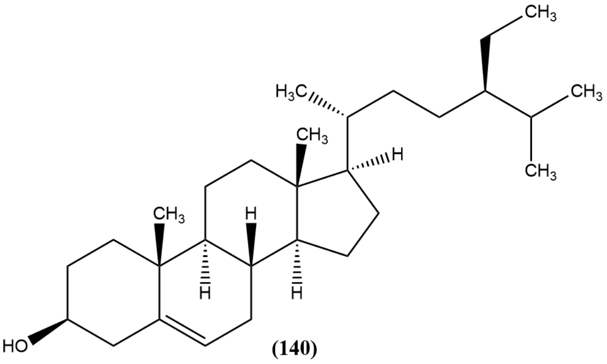

(140)

Figure S5. Triterpens and steroids identified in the species of the genus *Miconia* (Compounds 112-140).

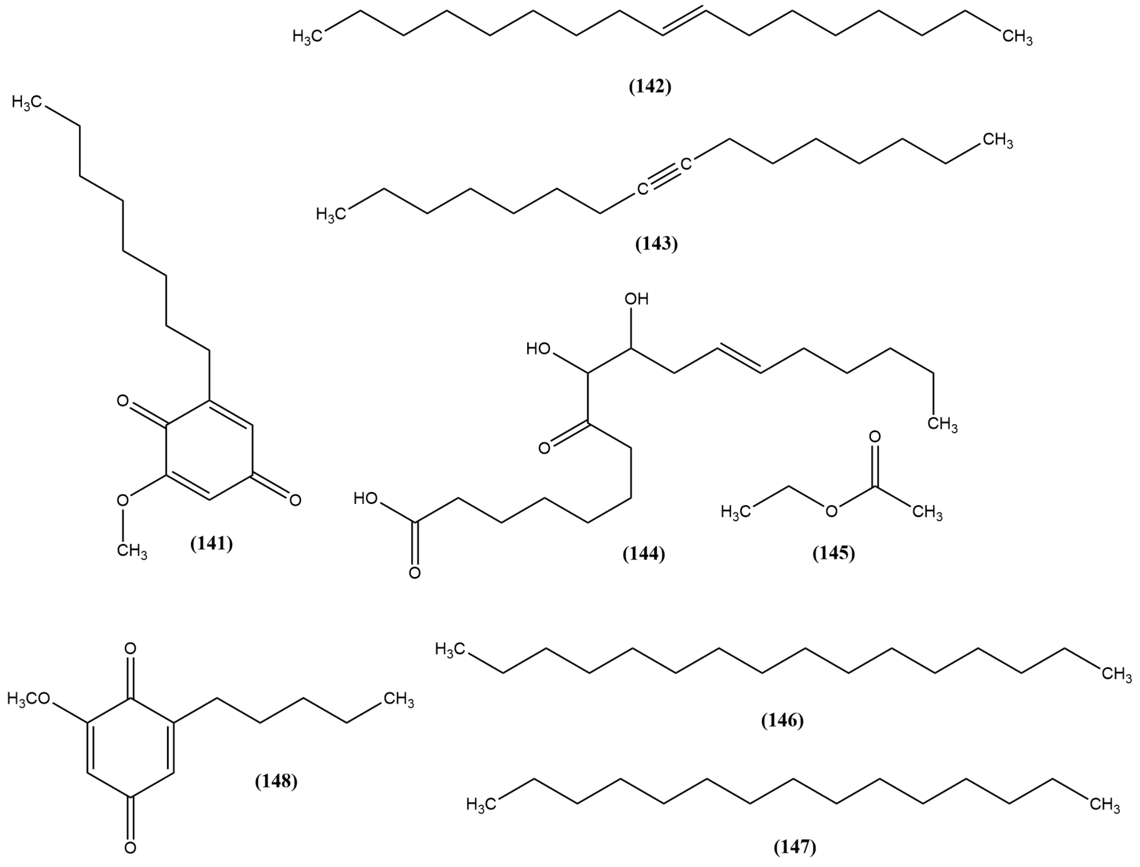

Figure S6. Other compounds identified in the species of the genus *Miconia* (Compounds 141-148).
